# Perceived stress and sickness absence: a prospective study of 17,795 employees in Denmark

**DOI:** 10.1007/s00420-019-01420-9

**Published:** 2019-02-27

**Authors:** Sannie Vester Thorsen, Jacob Pedersen, Mari-Ann Flyvholm, Jesper Kristiansen, Reiner Rugulies, Ute Bültmann

**Affiliations:** 1 The National Research Centre for the Working Environment (NRCWE), Lersø Parkallé 105, 2100 Copenhagen, Denmark; 2 Department of Health Sciences, Community and Occupational Medicine, University of Groningen, University Medical Center Groningen, Antonius Deusinglaan 1, 9713 AV Groningen, The Netherlands

**Keywords:** Stress symptom, Stress reaction, Sex difference, Short-term sickness absence, Long-term sickness absence

## Abstract

**Objectives:**

The aims were to examine (1) the prospective association between perceived stress and sickness absence, and if this association (2) differed by sex, and (3) was stronger when only long-term sickness absence (≥ 31 days) instead of all-length sickness absence (≥ 1 day) was included. Moreover, different cut-points for the length of the sickness absence periods were applied.

**Methods:**

We followed respondents (10,634 women and 7161 men) from the ‘Work Environment and Health in Denmark’ 2014-survey for up to 18 months in the ‘Register of Work Absences’ from Statistics Denmark. Perceived stress was measured by a single question: “In the last 2 weeks, how often have you felt stressed?” We used Cox-regression with repeated events, adjusted for age, sector, education, and previous sickness absence.

**Results:**

The hazard ratio (HR) for all-length sickness absence (≥ 1 day) for “Often/Always” stress compared to “Seldom/Never” stress was statistically significant among both men (HR = 1.25 [1.13–1.38]) and women (HR = 1.43 [1.34–1.51]). The HR was statistically significant for women (HR = 2.26 [1.89–2.70]), but not for men (HR = 1.22 [0.86–1.73]), when the analyses were restricted to long-term sickness absence (≥ 31 days). The sex-difference was statistically significant. Additional analyses with cut-points at ≥ 2, ≥ 4, ≥ 6, ≥ 8, ≥ 11, ≥ 15, ≥ 20, and ≥ 25 sickness absence days showed that among women, the HR increased gradually with increasing lengths of the sickness absence periods.

**Conclusions:**

The prospective association of perceived stress with risk of sickness absence was stronger among women than men. Among women, perceived stress was more strongly associated with long-term sickness absence than with all-length sickness absence.

## Introduction

Recent results from the 2016 Work Environment and Health in Denmark survey showed that 15.6% of the Danish working population reported ‘often’ or ‘always’ to having been stressed the last 2 weeks (Jensen et al. 2018). In Denmark ‘stress’ is one of the main reasons stated by long-term sick-listed employees, when asked about the cause of their sickness absence (Nielsen et al. 2010). Although the term ‘stress’ is commonly used, stress is neither a well-defined term nor a medical diagnosis. The term ‘stress’ has been used to describe working conditions (Jarvelin-Pasanen et al. 2018), the body’s reaction to stressors (Yang et al. 2015), or feelings of distress (Vitaliano et al. 1984). Okihiro et al. suggested to divide stress into three sub-categories: (a) stressors—negative events and conditions; (b) perceived stress—the subjective experience; and (c) stress symptoms—physiological and mental reactions (Okihiro et al. 2017). This article focuses on ‘perceived stress’, i.e., an individual’s own perception of his or her stress-level.

Several studies have examined the association of work stressors with sickness absence. For example job strain, effort–reward imbalance, and other adverse psychosocial working conditions have been associated with sickness absence in prospective studies (Head et al. 2007; Clausen et al. 2014; Trybou et al. 2014; Mortensen et al. 2016). Prospective studies have also shown an association between stress symptoms and sickness absence, e.g., burnout and fatigue have been associated with sickness absence (Bültmann et al. 2013; Salvagioni et al. 2017; Hoofs et al. 2017; Andersen et al. 2018). Perceived stress has been associated with different health outcomes, e.g., slower wound healing (Ebrecht et al. 2004) or increased risk of vascular diseases (Katsarou et al. 2013), but little is known about the prospective association between perceived stress and sickness absence. A study of 4114 male and female Danish public employees found that perceived stress predicted long-term sickness absence (≥ 21 days) with a hazard ratio of 1.33 [95% CI: 1.13–1.56] (Grynderup et al. 2016). Another study of 198 Swedish women visiting a health care center found that combined work-related and person-related perceived stress predicted sickness absence (≥ 8 days) with a relative risk of 4.34 [95% CI: 1.72–10.99] (Holmgren et al. 2013).

According to the allostatic load model, perceived stress may lead to an overstimulation where the adaptive systems are not efficiently turned on and off (McEwen 1998). This may result in illnesses such as headaches (Chrousos 2009), muscle aches (Chrousos 2009), weakening of the body’s immune system (Volmer and Fritsche 2016), exhaustion disorder (Grossi et al. 2015) or cardiovascular diseases (Kivimäki and Steptoe 2018). Perceived stress may also cause changes in behavior. An employee may refrain from taking sickness absence because he or she does not feel he or she can afford to be away from work. An indication of this mechanism has been observed in studies that show an increase in sickness presenteeism among employees with high perceived stress and high levels of job stressors (Elstad and Vabo 2008; Musich et al. 2006).

Even though studies of perceived stress and sickness absence are limited, we may assume such an association exists. It is also possible that the association varies with the length of the sickness absence periods. We hypothesize that perceived stress is primarily associated with long-term sickness absence, because short-term sickness absence may be cancelled out by behavioral mechanisms, i.e., an employee may go to work while sick. The association between perceived stress and sickness absence may also be different for men and women. Several studies indicate that women respond more strongly to stress than men (Matud 2004; Afifi 2007; Bale and Epperson 2015), which may lead to stronger associations of perceived stress with sickness absence among women. To the best of our knowledge, no studies have examined the associations of perceived stress by sex or with different lengths of sickness absence yet. It is relevant to examine these associations because it will add to the understanding of sickness absence from the labor market, and because it may add to the development of preventive measures.

In this study, we examined the prospective association between a one-item measure of perceived stress and register-based sickness absence. We investigated if the association (1) differed by sex and (2) was stronger for long-term sickness absence (≥ 31 days) than for all-length sickness absence (≥ 1 day). Moreover, different cut-points for the length of the sickness absence periods were applied.

## Methods

We linked the ‘Work Environment and Health in Denmark’ (WEHD) 2014-survey (The National Research Centre for the Working Environment 2015) with 18 months follow-up of sickness absence data from the Danish Register of Work Absence (Statistics Denmark 2016a).

### Perceived stress—The Work Environment and Health in Denmark (WEHD) survey

The Danish National Research Centre for the Working Environment has since 1990 conducted questionnaire surveys to measure work environment and health. The WEHD 2014-survey consisted of a cohort-sample (responders from a random baseline-sample in 2012, *N* = 15,852), and a 2014 random sample (*N* = 35,023). The invited individuals were employees, 18–66 years old, with a monthly income of minimum 3000 DKr/400 € and a minimum of 35 work hours per month during the last 3 months. The survey was web-based, non-responders received a reminder by phone and later a reminder with a paper-questionnaire. The response rate increased from 37% before the reminders to 57% after the last reminder (*N* responders = 29,166, web-based answers = 24,429, paper-questionnaire answers = 4737). A total of 27,246 individuals responded to the question on perceived stress (54%). The baseline-sample included 51% women, the mean age was 44 years, and 37% had a higher education. The sample of responders included 54% women, the mean age was 47 years, and 42% had a higher education. Among those who answered the stress-question, 54% was women, the mean age was 47 years, and 43% had a higher education. The wording of the stress-question was: “In the last 2 weeks, how often have you felt stressed?” with the response options: “Always”, “Often”, “Sometimes”, “Seldom”, “Never”.

### Sickness absence—The Danish Register of Work Absence

The Danish Register of Work Absence is a combination of Statistics Denmark’s ‘absence and employment’-register (FRAN) and ‘periods of absence’-register (FRPE). It has start- and end-dates of all absence periods due to ‘own sickness’, ‘child sickness’, ‘occupational injury’ and ‘maternity and adoption leave’ from (1) all public institutions, (2) all private companies with more than 250 employees, (3) a sample of private companies with 10–250 employees (a new sample drawn every year). Private companies with less than ten employees are not included (Statistics Denmark 2016b). The register covers 100% of all public employees and about 37% of all private employees. We were able to link 17,953 of the 27,246 WEHD-responders (66%) to the ‘Register of Work Absences’, that is, 66% of the responders worked in workplaces covered by the register. We used ‘own sickness absence’ as outcome. We excluded employees who were sickness absent at baseline (*N* = 158), leaving 17,795 individuals for the analyses. In the final sample 60% was women, the mean age was 47 years, and 50% had a higher education. Compared to the baseline-sample, the final sample consisted of significantly more women, had a higher mean age, and a higher education (tested by chi-square and *t* test). Employees were followed from response-date up to 18 months follow-up; the mean follow-up time was 15 months.

We defined the outcomes as all-length sickness absence, i.e., a sickness absence period of at least 1 day (≥ 1 day), and long-term sickness absence (≥ 31 days). Based on the Danish social security system we chose 31 days as the cut-point for long-term sickness absence, because employers are reimbursed for sickness absence periods longer than 30 days by the municipality (for all shorter periods the employer pays the employees’ sick-pay). Additionally, the outcome was defined as sickness absence periods of a minimum of 2, 4, 6, 8, 11, 15, 20, and 25 days.

### Covariates from the Central Person Register (CPR), the Population Education Register (BU), and the Danish Register of Work Absence

Covariates were obtained from the Central Person Register (CPR), the Population Education Register (BU), and the Danish Register of Work Absence. The covariates were: sex (male/female), age (in years), sector (private/state/municipality/region), education (Primary school or unknown education (unknown: *N* = 212 employees)/Upper secondary school/Professional internship, apprentice, trainee/1–3 years higher education/5 years higher education), and previous sickness (total absence days previous 2 months before baseline). The analyses included sector as a categorical variable, all other covariates were included as continuous variables.

### Statistical analysis

We used Cox-regression with a frailty model for repeated events (Christensen et al. 2007), i.e., we allowed for multiple sickness absence periods for the same employee. Employees were censored when lost to follow-up (e.g., when a new sample was drawn to the register *N* = 1266, when employees lost or changed job *N* = 1843, or went on maternity leave *N* = 197). First, we analyzed the association between perceived stress and all sickness absence periods (≥ 1 day), followed by analyses with long-term sickness absence as outcome (≥ 31 days). Second, the association between perceived stress and sickness absence periods of a minimum of 2, 4, 6, 8, 11, 15, 20, and 25 days were examined. All analyses were conducted separately for men and women, and were performed unadjusted and adjusted for age, sector, education, and previous sickness absence. We tested for multiplicative interaction between sex and perceived stress. Using visual inspection of the cumulative hazard plots, we found the proportional hazard assumption fulfilled for all analyses, except for the analysis for men with ‘Sometimes’-stress and long-term sickness absence and consequently we did not report the results from this analysis. Finally, we performed sensitivity analyses with employees with unknown education (*N* = 212) excluded.

### Ethics

The WEHD survey was approved by the Danish Data Protection Agency, reference number 2012-54-0017. According to Danish law, questionnaire-based and register-based studies do not need approval by committees of ethics, nor do they need informed consent (The Committee on Biomedical Research Ethics 2011; The Danish Data Protection Agency 2010).

## Results

Among 17,795 employees, 18% women and 12% men reported ‘Often/Always’-stress for the last 2 weeks. A total of 89.5% of all sickness absence periods were short-term periods of 1–7 days; 3.8% were long-term sickness absence periods of ≥ 31 days (Table 1).

**Table 1:** Sample characteristics (*N* = 17,795)

	Women			Men			Total		
	*N*	(%)	Mean	*N*	(%)	Mean	*N*	(%)	Mean
Women and men included in the study	10,634			7161			17,795		
Perceived stress last 2 weeks									
Often/always	1876	17.6		871	12.2		2747	15.4	
Sometimes	5508	51.8		4491	62.7		9999	56.2	
Seldom/never	3250	30.6		1799	25.1		5049	28.4	
Number of sickness absence periods in the entire sample									
1–7 day periods	35,545	89.3		16,378	90.1		51,923	89.5	
8–30 day periods	2605	6.5		1266	7.0		3871	6.7	
31 + day periods	1657	4.2		539	3.0		2196	3.8	
Total number of periods	39,807			18,183			57,990		
Age (in years)	10,634		47	7161		47	17,795		47
Follow-up time (in months)	10,634		15.7	7161		15.2	17,795		15.5
Previous sickness absence last 2 months (in days)	10,634		2.1	7161		1.1	17,795		1.7
Sector									
Private	3034	28.5		4342	60.6		7376	41.5	
State	1183	11.1		1173	16.4		2356	13.2	
Municipality	5007	47.1		1310	18.3		6317	35.5	
Region	1410	13.3		336	4.7		1746	9.8	
Education									
Primary school/unknown	1162	10.9		1011	14.1		2173	12.2	
Upper secondary school	631	5.9		467	6.5		1098	6.2	
Professional internship/apprentice/trainee	3223	30.3		2408	33.6		5631	31.6	
1–3 years higher education	4250	40.0		1986	27.7		6236	35.0	
5 years higher education	1368	12.9		1289	18.0		2657	14.9	

### Perceived stress and risk of all-length and long-term sickness absence

The unadjusted and the adjusted hazard ratios of the association of perceived stress with all-length and long-term sickness absence are shown in Tables 2 and 3, respectively. The result pattern was similar for the unadjusted and the adjusted analyses.

**Table 2:** Unadjusted hazard ratios for all-length sickness absence periods (≥ 1 day) and for long-term sickness absence (≥ 31 days)

Perceived stress	All-length sickness absence periods (periods ≥ 1 day)			Long-term sickness absence (periods ≥ 31 days)		
	Hazard ratio	95% confidence interval	*p* value	Hazard ratio	95% confidence interval	*p* value
Women						
Often/always-stress	1.52	[1.43–1.61]	< 0.0001	2.51	[2.10-3.00]	< 0.0001
Sometimes-stress	1.21	[1.15–1.27]	< 0.0001	1.49	[1.25–1.76]	< 0.0001
Seldom/never-stress (reference level)	1	–	–	1	–	–
Men						
Often/always-stress	1.32	[1.19–1.46]	< 0.0001	1.35	[0.97–1.90]	0.08
Sometimes-stress	1.17	[1.08–1.26]	< 0.0001	*	*	*
Seldom/never-stress (reference level)	1	–	–	1	–	–
Total						
Interaction sex and often/always-stress			0.019			0.001
Interaction sex and sometimes-stress			0.42			*

*We did not perform  the analyses of ‘Sometimes’-stress and ‘long-term sickness absence’ for men because the proportional hazard assumption was not fulfilled

**Table 3:** Adjusted hazard ratios for all-length sickness absence periods (≥ 1 day) and for long-term sickness absence (≥ 31 days), covariates were age, sector, education, and previous sickness absence

Perceived stress	All-length sickness absence periods (periods ≥ 1 day)			Long-term sickness absence (periods ≥ 31 days)		
	Hazard ratio	95% confidence interval	*p* value	Hazard ratio	95% confidence interval	*p* value
Women						
Often/always-stress	1.43	[1.34–1.51]	< 0.0001	2.26	[1.89–2.70]	< 0.0001
Sometimes-stress	1.18	[1.13–1.24]	< 0.0001	1.46	[1.23–1.73]	< 0.0001
Seldom/never-stress (reference level)	1	–	–	1	–	–
Men						
Often/always-stress	1.25	[1.13–1.38]	< 0.0001	1.22	[0.86–1.73]	0.26
Sometimes-stress	1.16	[1.07–1.25]	0.0002	*	*	*
Seldom/never-stress (reference level)	1	–	–	1	–	–
Total						
Interaction sex and often/always-stress			0.049			0.002
Interaction sex and sometimes-stress			0.54			*

*We did not perform the analyses of ‘Sometimes’-stress and ‘long-term sickness absence’ for men because the proportional hazard assumption was not fulfilled

Table 3 shows that women reporting ‘Often/Always’-stress and ‘Sometimes’-stress had a significantly higher risk of both ‘all-length sickness absence’ (≥ 1 days) and ‘long-term sickness absence’ (≥ 31 days) compared to the reference group of ‘Never/Seldom’-stress. The hazard ratio (HR) increased from women reporting ‘Sometimes’-stress to women reporting ‘Often/Always’-stress. Moreover, the HR of the association of perceived stress with ‘long-term sickness absence’ (≥ 31 days) was significantly higher than the HR for ‘all-length sickness absence’. Men reporting ‘Often/Always’-stress and ‘Sometimes’-stress had a significantly higher risk of ‘all-length sickness absence’ compared to the reference group of ‘Never/Seldom’-stress. The risk for ‘long-term sickness absence’ was not significantly higher for men reporting ‘Often/Always’-stress compared to the reference group of ‘Never/Seldom’-stress.

The HR was generally higher for women than men. The *p* value for the interaction term between sex and perceived ‘Often/Always’-stress was 0.049 for ‘all-length sickness absence’ and 0.002 for ‘long-term sickness absence’, i.e., a statistically significant difference between women and men.

The sensitivity analyses where we excluded employees with unknown education showed similar results (results not shown).

### Perceived stress and different cut-points for the lengths of the sickness absence periods

**Fig. 1:**
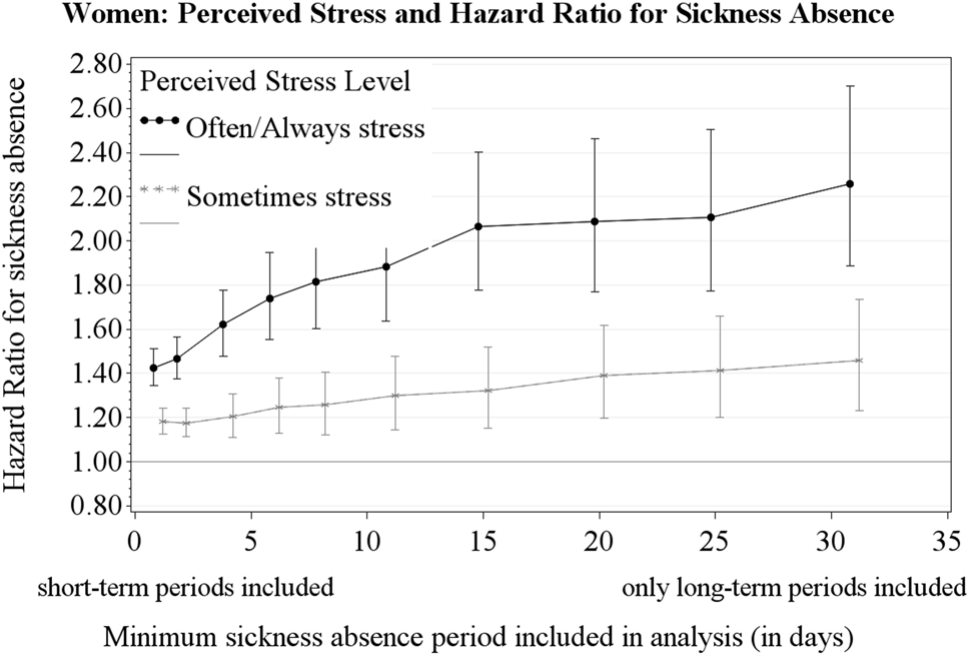
The hazard ratio of perceived stress for sickness absence (‘Often/Always’-stress, ‘Sometimes’-stress, versus reference level ‘Seldom/Never’-stress). Results shown for sickness absence defined as periods ≥ 1, ≥ 2, ≥ 4, ≥ 6, ≥ 8, ≥ 11, ≥ 15, ≥ 20, ≥ 25, and ≥ 31 days

**Fig. 2:**
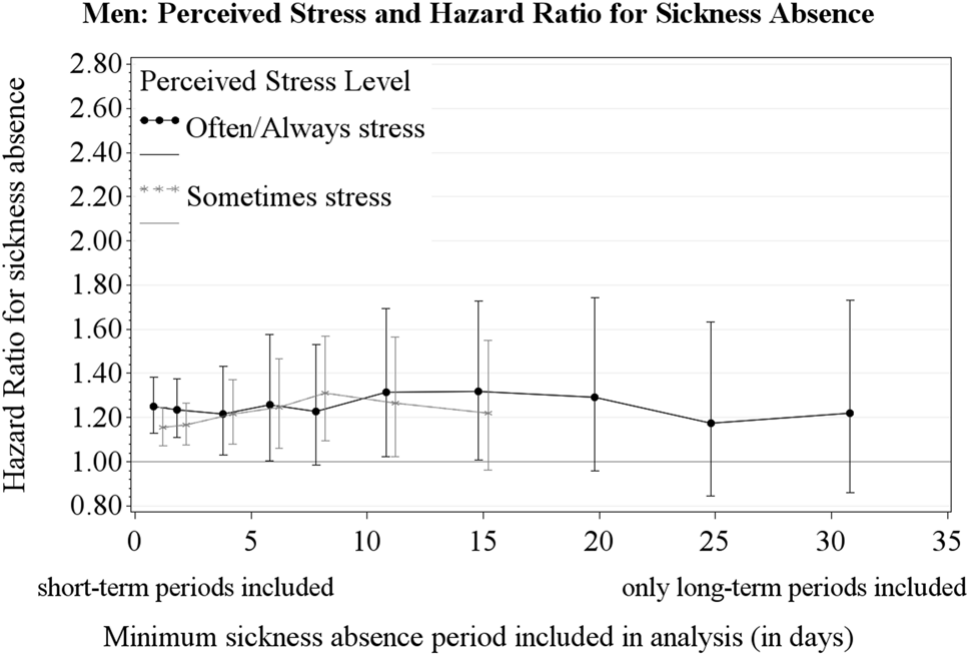
The hazard ratio of perceived stress for sickness absence (‘Often/Always’-stress, ‘Sometimes’-stress, versus reference level ‘Seldom/Never’-stress). Results shown for sickness absence defined as periods ≥ 1, ≥ 2, ≥ 4, ≥ 6, ≥ 8, ≥ 11, ≥ 15, ≥ 20, ≥ 25, and ≥ 31 days. The analyses for ‘Sometimes’-stress and sickness absence ≥ 20, ≥ 25, and ≥ 31 days were not conducted because the proportional hazard assumption was not fulfilled

Figures 1 and 2 show the HRs for perceived stress and different cut-points for the length of sickness absence periods (≥ 1, ≥ 2, ≥ 4, ≥ 6, ≥ 8, ≥ 11, ≥ 15, ≥ 20, ≥ 25 and ≥ 31 days) for women and men, respectively. Figure 1 shows a dose–response relationship with sickness absence for the frequency of perceived stress among women (i.e., from ‘Seldom/Never’ to ‘Sometimes’ to ‘Often/Always’-stress). Figure 1 also illustrates that the HR for women with ‘Often/Always’-stress increases gradually when short-term sickness absence periods were omitted from the analyses. Figure 2 shows the no dose–response relationship among men (i.e., ‘Sometimes’-stress and ‘Often/Always’-stress have approximately equal HR) and that the HR for men did not increase when short-term sickness absence periods were omitted from the analyses.

## Discussion

Perceived ‘Often/Always’-stress was statistically associated with all-length sickness absence (≥ 1 day) for both women and men, while the association of perceived stress with long-term sickness absence (≥ 31 days) was significant for women only. Among women, the HR for long-term sickness absence was higher than the HR for all-length sickness absence. Moreover, in women a gradual increase of HR was observed with longer sickness absence periods.

The study revealed a significant sex-difference in the association between perceived stress and sickness absence. The association was higher among women than men, in particular for long-term sickness absence. A few prospective studies have shown associations between perceived stress and sickness absence (Holmgren et al. 2013; Grynderup et al. 2016), but no previous studies have to our knowledge examined sex-differences. Studies have shown that more women than men report stress (Matud 2004; Jensen et al. 2018), that women in general rate their health lower than men (Singh-Manoux et al. 2008), and women have more sickness absence than men (Akerlind et al. 1996; Gimeno et al. 2004; Thorsen et al. 2016). Studies of work stressors and sickness absence have examined sex-differences, but the results are not consistent and not specific for perceived stress (Lund et al. 2005; Nielsen et al. 2006; Head et al. 2007; Mortensen et al. 2016).

The sex-difference in our results may be related to stronger reactions to perceived stress in women compared to men. Studies have shown that women and men have different stress responses (Kajantie and Phillips 2006), and studies have proposed that those differences may be caused by socialized behaviors (Matud 2004; Afifi 2007) or by hormone and genetic differences (Bale and Epperson 2015). However, there may be other explanations for our results. It is possible that men more often have positions and jobs in companies where sickness absence due to stress is not tolerated, i.e., they may be laid off if they show early signs of stress symptoms, which may play into a healthy worker effect. Moreover, women may also be more inclined to report stress than men, and men may more often be in denial. To what extent these possible mechanisms contribute to the sex differences in the association between perceived stress and sickness absence remains to be clarified.

For women, the study revealed a stronger association of perceived stress with long-term sickness absence compared to all-length sickness absence. Analyses with all-length sickness absence were dominated by sickness absence periods of shorter length, since short-term sickness absence was much more frequent than long-term sickness absence (89.5% were 1–7 day periods versus 3.8% were 31 + day periods). For women, the HR for long-term sickness absence was substantially higher than the HR for all-length sickness absence, i.e., these results indicate that perceived stress increased the risk for long-term sickness absence more than the risk for short-term sickness absence. We know of no other studies that examine perceived stress and different lengths of sickness absence periods. Some studies of work stressors have examined sickness absence periods of different lengths and have found a tendency for stronger associations with long-term sickness absence than with short-term sickness absence (Allebeck and Mastekaasa 2004; Nielsen et al. 2006). Those studies point in the same direction as ours, but they did not examine perceived stress, which hinders a direct comparison with our study.

Several strengths and limitations must be addressed. A strength of this study is that we included a large sample of the working population, with employees from both the private and the public sector, and with high and low educational level. Moreover, data were linked with a national register (the Danish register of Work absence), that provided information on sickness absence periods of all lengths. Even though our sample is not a representative sample of the Danish population, due to non-response and since the register of work absence does not cover all employees, the sample still represents a wide variety of Danish employees. It is, therefore, likely the results are generalizable to the Danish workforce. A limitation is that we measured perceived stress with only one question and at only one point in time, asking about the last 2 weeks. One question at one point in time may not capture the complexity of stress. More questions at several time points would provide a more in-depth understanding of the association. However, previous studies have found a one-question measure of stress to be adequate for group level analyses (Elo et al. 2003; Lindegard et al. 2014). It should also be noted that reversed causality cannot be excluded. Previous health problems might be the reason both for perceived stress and later sickness absence. In this case, perceived stress is a predictor of later sickness absence, but it is not the cause. We have adjusted for the last 2 months of sickness absence, but this might not be sufficient to avoid reverse causality. Finally, we do not know the medical diagnosis of the sickness absence periods.

The associations between perceived stress and sickness absence are relevant on a population level and may add to further understanding of sickness absence differences for women and men. Moreover, our study provides suggestive evidence that preventive measures for perceived stress may reduce short-term sickness absence among both sexes, and long-term sickness absence among women.

## Conclusion

This study found statistically significant differences between women and men regarding the prospective association of perceived stress with sickness absence, with a clear dose–response relationship in women only. In women, perceived stress was more strongly associated with long-term sickness absence than all-length sickness absence.
